# Investigating changes in the blood-brain barrier in schizophrenia spectrum disorders: design and methodology of a longitudinal dynamic contrast-enhanced MRI study

**DOI:** 10.1007/s00406-026-02316-9

**Published:** 2026-08-04

**Authors:** Anne Wendl, Amelie Zöllinger, Daniel Lukas, Isabel Lutz, Michelle Schamberger, Constanze Lobinger, Isabel Maurus, Alkomiet Hasan, Elias Wagner, Peter Falkai, Andrea Schmitt, Florian J. Raabe, Kolja Schiltz, Klaus Seelos, Sophia Stöcklein, Emanuel Boudriot, Vladislav Yakimov, Lukas Roell, Daniel Keeser, Joanna Moussiopoulou

**Affiliations:** 1 Department of Psychiatry and Psychotherapy, LMU University Hospital, LMU Munich, Munich, Germany; 2 International Max Planck Research School for Translational Psychiatry (IMPRS-TP), 80804 Munich, Germany; 3 NeuroImaging Core Unit Munich (NICUM), LMU University Hospital, LMU Munich, 80336 Munich, Germany; 4 Department of Psychiatry, Psychotherapy and Psychosomatics, Faculty of Medicine, University of Augsburg, 86156 Augsburg, Germany; 5 German Center for Mental Health (DZPG), partner site Munich, Augsburg, Germany; 6 Evidence-based Psychiatry and Psychotherapy, Faculty of Medicine, University of Augsburg, Stenglinstrasse 2, 86156 Augsburg, Germany; 7 Max Planck Institute of Psychiatry, 80804 Munich, Germany; 8 Laboratory of Neuroscience (LIM27), Institute of Psychiatry, University of Sao Paulo, São Paulo, Brazil; 9 Institute of Neuroradiology, LMU University Hospital, LMU Munich, Marchioninistr. 15, 81377 Munich, Germany; 10 Department of Radiology, LMU University Hospital, LMU Munich, Munich, Germany

**Keywords:** Schizophrenia spectrum disorders, Blood–brain barrier, Dynamic contrast-enhanced MRI, Inflammation, Deep phenotyping

## Abstract

Schizophrenia spectrum disorders (SSDs) are clinically and biologically heterogeneous and lack reliable biomarkers for stratification, treatment response and course prediction. Evidence from postmortem, fluid biomarker, and neuroimaging studies suggests that changes in the blood–brain barrier (BBB) may contribute to pathophysiology in a biologically defined subgroup. However, findings in this context are inconsistent and often based on cross-sectional or indirect measures. This study is a longitudinal, multimodal investigation designed to quantify BBB permeability across disease phases using dynamic contrast-enhanced magnetic resonance imaging (DCE-MRI) and to integrate these measures with deep clinical phenotyping. We recruit inpatients with SSDs and healthy controls (HC). Participants undergo multimodal MRI including DCE-MRI at three time points: acute psychosis (baseline; V1), early treatment (4–6 weeks; V2), and long-term follow-up (2.5 years; V3). Clinical characterization includes standardized measures of psychopathology, functioning and cognition. Blood samples are collected at each visit, while CSF is obtained at V1. DCE-MRI-derived voxel-wise permeability metrics are then analyzed. Primary objectives are to compare BBB leakage cross-sectionally between SSD and HC. Secondary objectives include (i) characterizing spatial and temporal leakage profiles across illness phases, (ii) analyzing associations with psychopathology and biological (e.g., inflammatory) signatures, as well as exploratory identification of subgroups. By providing a longitudinal, BBB-specific neuroimaging framework embedded in a deep phenotyping infrastructure, the IMPACT study aims to elucidate BBB alterations in SSDs and to support stratification approaches in precision psychiatry.

## Introduction

People with schizophrenia spectrum disorders (SSDs) show heterogeneity in symptom profiles, development of functional capacity, and responsiveness to treatment, ranging from isolated acute episodes to chronic, treatment-resistant illness [1]. Despite decades of research into neuroimaging, fluid biomarkers and genetics, reliable biological markers for diagnosis or treatment guidance are still lacking and the underlying pathophysiology remains incompletely understood [2]. Amongst emerging biological systems of interest, the blood-brain barrier (BBB) gains greater recognition [3]. As a highly selective interface formed by endothelial cells, pericytes, and astrocytic end-feet, the BBB strictly regulates molecular and cellular exchange between the systemic circulation and the central nervous system (CNS) [4]. Structural or functional changes in this barrier can permit entry of peripheral inflammatory mediators or immune cells into brain tissue, potentially initiating neuroinflammatory and degenerative processes implicated in neuropsychiatric disorders [5]. Also, vascular alterations hindering BBB integrity and angiogenesis appear to be more pronounced in individuals with high levels of inflammation, suggesting that inflammatory processes may contribute to endothelial dysfunction and reduced neurogenesis [6]. Although BBB disturbance is well documented in neurological disorders such as Alzheimer’s disease, Parkinson’s disease, and multiple sclerosis [7], its role in SSDs remains only partially elucidated.

Evidence from postmortem analyses and in vivo assessments of blood and cerebrospinal fluid (CSF) parameters been linked to alterations of the BBB in SSDs.

Postmortem investigations reveal structural and morphological changes within the neurovascular unit [8], including reduced numbers of pericapillary oligodendrocytes [9], endothelial vacuolation, thickening of the basal membrane, and hypertrophy of astrocytic end-feet [10]. However, these data are restricted by small sample sizes, potential biases related to the cause of death, and the inability to capture in vivo or longitudinal dynamics in BBB changes. Fluid-based biomarkers such as the CSF to serum albumin ratio (Q_Alb_), widely regarded as a state-of-the-art method for assessing BBB dysfunction [3], have been reported to be elevated in individuals with SSDs [11, 12]. However, this marker predominantly reflects the function of the blood-CSF barrier (BCSFB) rather than the BBB, and is susceptible to extracerebral influences and other confounding factors [13]. Similarly, recent studies reporting elevated circulating endothelial glycocalyx components in antipsychotic-naïve first-episode psychosis suggest endothelial damage and possible BBB compromise [14], yet such peripheral biomarkers cannot directly quantify and localize regional BBB permeability in vivo, underscoring the need for specific imaging approaches.

Dynamic contrast-enhanced MRI (DCE-MRI) is a highly sensitive technique for quantifying even subtle BBB leakage and has demonstrated utility across various neuropsychiatric conditions, including Alzheimer’s disease [15, 16]. BBB disruption can be measured by the extravasation of an intravenously administered contrast agent (CA), resulting in image contrast enhancement [17]. Temporal changes in T1-weighted signal intensity are analyzed using pharmacokinetic models to derive quantitative permeability metrics [18, 19]. The most widely applied parameter, K_trans_, reflects the rate of CA transfer from plasma to the extravascular space [17, 18]. One cross-sectional DCE-MRI study that has assessed BBB permeability in vivo in SSD patients reported increases in K_trans_ values within the thalamus in a cohort of 29 patients compared with 18 healthy controls, but its statistical power was limited due to the small sample size [20]. Consequently, this finding requires replication and more detailed characterization of the affected phenotype. Moreover, the cross-sectional design precludes insight into the temporal dynamics of BBB alterations across stages of illness [3, 20].

A more comprehensive, systematic approach is therefore needed to investigate BBB integrity in SSD and to relate it to clinical phenotype, illness stage, and biologically informed profiles, including peripheral and cerebrospinal fluid markers [3, 21]. Such efforts are essential to establish the clinical relevance of BBB dysfunction, identify affected patient subgroups, and evaluate its potential as a target for precision psychiatry and individualized therapeutic interventions [3, 22].

The *Imaging-Blood-Brain-Barrier-Permeability-in-Schizophrenia-Spectrum-Disorders-Study* (IMPACT Study) is a longitudinal, multimodal, naturalistic investigation of BBB function in SSDs. DCE-MRI is performed at multiple illness and treatment stages to assess spatial and temporal patterns of BBB permeability [15, 18], analyzed via pharmacokinetic modeling [19, 23]. Combined with clinical, cognitive, cerebrospinal fluid, and blood biomarkers, as well as additional neuroimaging modalities, this design enables comprehensive biological phenotyping. This study aims to (i) compare BBB leakage between individuals with SSDs and healthy controls, (ii) characterize its spatial and temporal profile, (iii) examine clinical correlates, including symptoms, cognition, and psychosocial functioning, (iv) investigate underlying molecular mechanisms, and (v) evaluate BBB dysfunction as a biomarker for SSD subgroups. Ultimately, IMPACT seeks to advance biomarker-guided, mechanistically informed precision psychiatry [3].

## Methods and design

### Study design and setting

IMPACT is a prospective, naturalistic, longitudinal observational investigation conducted at the Department of Psychiatry and Psychotherapy, LMU University Hospital Munich and the University of Augsburg, with harmonized procedures embedded in the *Clinical Deep Phenotyping of Treatment Response in Schizophrenia (CDP-STAR)* infrastructure [24]. CDP-STAR was approved by the local ethics committee (project number 24–0341, 2024) and is registered at the German Clinical Trials Register (DRKS00034820) and OSF (https://doi.org/10.17605/OSF.IO/SQ2TZ). The IMPACT protocol was independently approved by the local ethics committee of LMU Munich (reference number 21-1139) and is conducted in accordance with the Declaration of Helsinki.

### Study population

#### Schizophrenia spectrum disorder group

The study includes inpatients diagnosed with schizophrenia spectrum disorders (SSDs), according to the criteria of the Diagnostic and Statistical Manual of Mental Disorders (DSM-5-TR, Version 7.0.2). All diagnoses are confirmed using the Mini-International Neuropsychiatric Interview (M.I.N.I.) [25]. Additional eligibility criteria include an age range of 18 to 65 years, sufficient German language proficiency, the capacity to provide written informed consent, and inclusion in the Munich Mental Health Biobank (MMHB) [26]. Exclusion criteria comprise a primary psychiatric diagnosis other than SSDs, the presence of acute risk to self or others (e.g., acute suicidality), ongoing coercive treatment, or involuntary hospitalization at the time of study inclusion. Participants are also excluded if they are unable to provide informed consent, are currently pregnant, or have relevant comorbid disorders affecting the central nervous or immune system, such as epilepsy, dementia, multiple sclerosis, organic psychotic or organic affective disorders, or acute inflammatory conditions (e.g., acute respiratory tract infection), These comorbidities are assessed by an experienced physician based on self-report, review of clinical reports and overall clinical evaluation. Further reasons for exclusion are general contraindications for cranial MRI, such as implanted pacemakers, as well as specific contraindications to dynamic contrast-enhanced MRI (DCE-MRI), including acute renal failure or known hypersensitivity to Gadolinium-based contrast agents. Potential participants are routinely screened for eligibility by trained study physicians at our clinic, and written informed consent is obtained before the initiation of any study-related procedures.

#### Healthy control group

Healthy controls (HC) are recruited and screened to exclude current or past psychiatric disorders (as reported by the subject), relevant neurological diseases, MRI contraindications, and contraindications to gadolinium administration. Additional exclusion criteria include current use of psychotropic medication. The HC group participates only in a single study visit.

### Study timeline

**Fig. 1:**
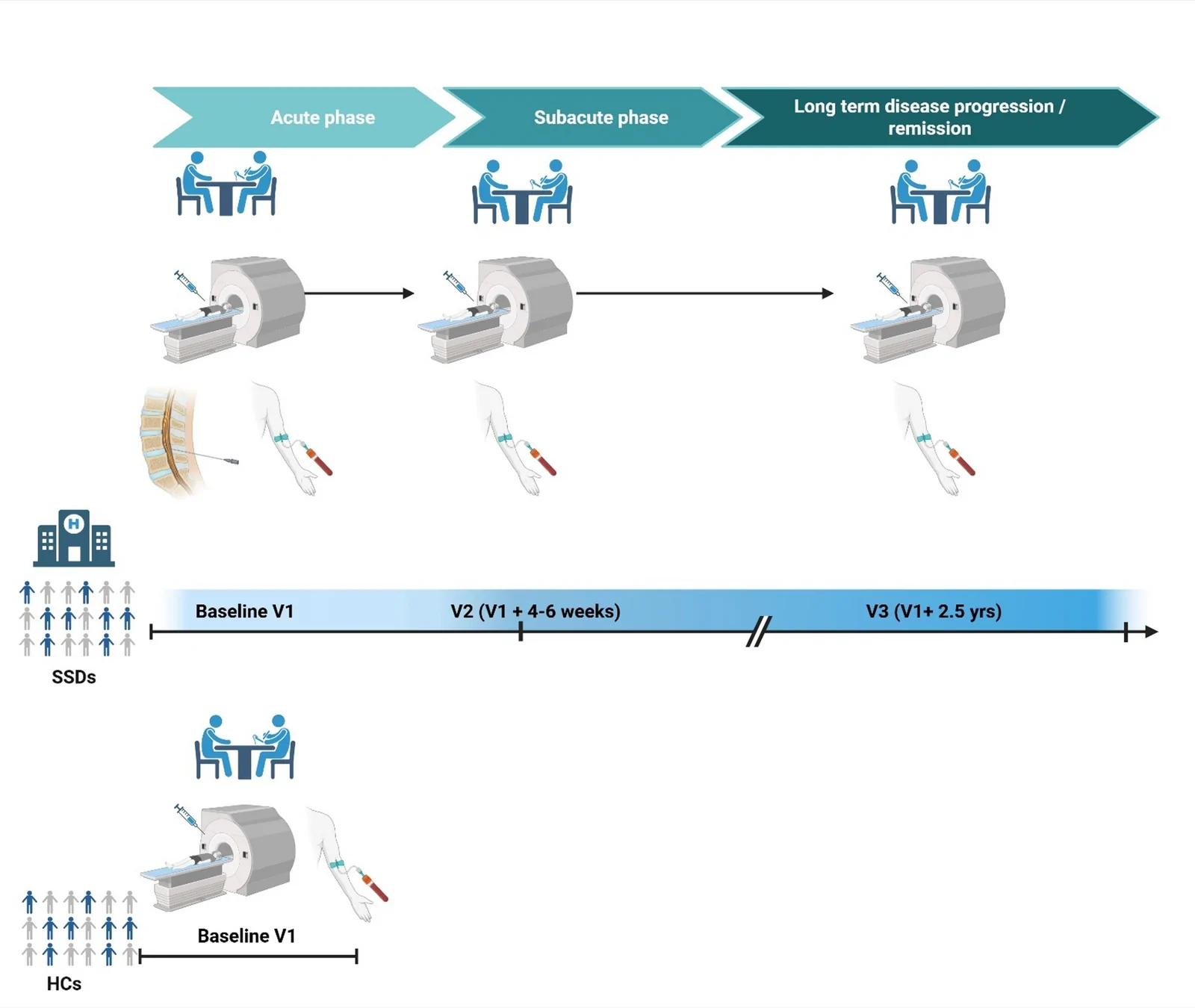
Study design and timeline

The study protocol is outlined in Fig. 1. Healthy controls participate in a single study visit, whereas individuals with SSD are invited to complete all three study visits. At baseline (visit 1; V1), all participants undergo a standardized assessment battery comprising multimodal MRI, including DCE-MRI, comprehensive clinical characterization, and venous blood sampling. Cerebrospinal fluid (CSF) is additionally collected via lumbar puncture in the SSD group when clinically indicated, in accordance with German guidelines [27]. Follow-up assessments of the SSD group are scheduled at 4–6 weeks (V2) and 2.5 years (V3) following baseline. These follow-up visits include multimodal and DCE-MRI, clinical and cognitive evaluations, and blood sampling to assess longitudinal changes across the acute phase, early treatment response, and long-term progression of illness.

### Clinical characterization

All participants (SSDs and HCs) complete structured interviews and standardized questionnaires to obtain comprehensive demographic and clinical information. This includes age, sex, height, and weight (used to calculate body mass index, BMI), educational background, and substance use. Whenever possible, self-reports are corroborated with available medical records to enhance data validity.

The clinical protocol also captures past and present somatic illnesses, with particular attention to neurological and inflammatory disorders and cardiometabolic risk factors, including smoking status, dyslipidemia, and obesity, which are known to contribute to the environmental risk burden in schizophrenia [28].

Participants in the SSD group additionally provide information on current pharmacological treatment and history of electroconvulsive therapy. Further illness-related variables include lifetime exposure to antipsychotic medication (quantified in months, chlorpromazine equivalents ), according to the *Defined Daily Dose* method [29]), duration of illness (defined as time since first psychiatric diagnosis), age at symptom onset, duration of untreated psychosis, number of psychiatric hospitalizations, and illness stage (first-episode vs. multi-episode psychosis).

Diagnostic validation and psychopathological characterization in individuals with SSDs are conducted at the baseline assessment using the Mini-International Neuropsychiatric Interview (M.I.N.I.) based on DSM-5 criteria, which is a validated and efficient tool for structured psychiatric diagnosis in clinical and research settings [25]. Symptom severity and global clinical status are assessed at all three visits using the Positive and Negative Syndrome Scale (PANSS) [30], the Calgary Depression Scale for Schizophrenia (CDSS) [31], the Clinical Global Impression scale (CGI) [32] and the Global Assessment of Functioning (GAF) scale [33]. In addition, participants complete the abbreviated version of the World Health Organization Quality of Life Instrument (WHOQOL-BREF) [34] as part of the V1 and V2 to assess health-related quality of life.

### Cognitive Testing

Cognitive performance is assessed in both the SSD and control groups using a standardized test battery administered by trained mental health professionals. To minimize practice effects, different test components are implemented at each timepoint (V1, V2, V3). At baseline (V1), the full battery, comprising the Brief Assessment of Cognition in Schizophrenia (BACS), the Trail Making Test (TMT), and the Montreal Cognitive Assessment (MoCA), is administered. At V2 (4–6 weeks), cognitive assessment is limited to the TMT to reduce retest effects, while at V3 both MoCA and TMT are repeated.

The TMT evaluates multiple cognitive domains commonly affected in individuals with SSDs, including attention, visual search and scanning, processing speed, task switching, cognitive flexibility, and executive function [35]. The BACS [36], a validated instrument developed specifically for patients with psychotic disorders, assesses six cognitive domains: verbal memory, working memory, motor speed, attention, executive function, and verbal fluency. These domains align with those identified as central by the Measurement and Treatment Research to Improve Cognition in Schizophrenia (MATRICS) Neurocognition Committee for evaluating cognitive deficits in schizophrenia [37]. In addition, the MoCA is employed to provide a broader evaluation of global cognitive functioning [38]. It assesses a wide range of cognitive abilities, including attention and concentration, executive functions, memory, language, visuospatial abilities, conceptual reasoning, calculations, and orientation.

### Multimodal brain imaging

All MRI data are acquired at the Department of Psychiatry and Psychotherapy, University Hospital of LMU Munich, using a 3-Tesla Magnetom Prisma scanner (Siemens Healthcare GmbH, Erlangen, Germany) equipped with a 64-channel head coil. Multimodal MRI (mMRI) acquisition follows the protocol of the Human Connectome Project (HCP) [39], adapted for use in psychiatric populations. Anatomical imaging includes high-resolution T1-weighted magnetization-prepared rapid acquisition gradient echo (T1-MPRAGE) and T2-weighted sampling perfection with application-optimized contrasts using different flip angle evolution (T2-SPACE). Functional imaging includes resting-state functional MRI (fMRI) using a gradient-echo echo-planar imaging sequence in anterior-posterior orientation as well as arterial spin labeling (ASL) for quantitative assessment of cerebral perfusion without the use of contrast agents. DCE-MRI, which forms an integral part of the multimodal MRI protocol, is described in detail in a separate section.

An experienced neuroradiologist reviews all MRI datasets. In cases where structural abnormalities are identified, participants are informed accordingly and receive appropriate clinical care, and their scans are excluded from further analysis.

#### Dynamic contrast-enhanced magnetic resonance imaging (DCE-MRI)

DCE-MRI is included in the multimodal imaging protocol to assess BBB integrity across multiple brain regions. The technique allows quantification of BBB permeability by measuring contrast agent efflux from the vasculature into brain tissue, which is then used to calculate whole-brain voxelwise leakage. The sequence has a total acquisition time of 23 min and a voxel size of 1.8 × 1.8 × 1.8 mm. A gadolinium-based contrast agent (Gadobutrol, Gadovist^®^, Bayer AG, Leverkusen, Germany) is administered intravenously via the antecubital vein at a dose of 0.1 mmol/kg body weight. Injection occurs at time point 12/80 of the scan, approximately 2 min after sequence onset, at a rate of 3 ml/s, followed by a 25–30 ml saline flush.

### Biobanking of blood and cerebrospinal fluid

All participants need to provide written informed consent for inclusion in the Munich Mental Health Biobank, which provides the infrastructure for our sample biobanking as previously described [3]. An overview of all conducted examinations can be found in Table 1. All individuals with SSDs undergo basic blood tests, including complete blood count analyses, as part of the clinical routine in our clinic (collected around 8 a.m.). At baseline (visit 1) the Psychiatry Biobank Blood Kit is collected, containing the following components: 1 × S-Monovette^®^ RNA exact (Sarstedt, Cat. No. 01.2048.001) for RNA extraction, 1 × 7.5 ml K3EDTA tube (Sarstedt, Cat. No. 01.1605.001) for DNA extraction, 1 × 9 ml K3EDTA tube (Sarstedt, Cat. No. 02.1066.001) for plasma-based analysis and 1 × 9 ml tube with coagulation activator (Sarstedt, Cat. No. 02.1063.001) for serum-based analysis. During the DCE-MRI scans, one additional 7,5 ml serum tube is collected via the peripheral venous catheter used for contrast agent administration.

As part of standard SSD diagnostics in Germany, CSF examinations are offered to individuals with SSD, in accordance with the national schizophrenia guidelines [40]. Q_Alb_ (as a continuous variable) was selected as indicative for CSF pathology or BCSFB disruption and adjusted for age according to the formula: Q_Alb_ = (4 + age/15) × 10 − 3 [41], age indicated as years. In all cases, CSF and serum are tested for neuronal autoantibodies to rule out autoimmune-mediated psychosis or encephalitis. These tests are performed in the laboratory of our hospital by a cell-based assay from EUROIMMUN (Lübeck, Germany) for autoimmune encephalitis (antibodies tested: NMDAR, α-amino-3-hydroxy-5-methyl-4-isoxazolepropionic acid receptor-1 (AMPAR-1), α-amino-3-hydroxy-5-methyl-4-isoxazolepropionic acid receptor-2 (AMPAR-2), CASPR2, LGI1 and γ-amino-butyric acid receptor B1/B2). Long-term storage is performed at − 80 °C.

Planned analyses of the biosamples include:

- *Inflammatory profiling*: quantification of peripheral and CSF inflammatory markers (e.g., IL-6, TNF-α, IL-1β, CRP) using multiplex immunoassays and correlation with regional BBB leakage (K^trans^).
- *Blood–CSF barrier markers*: determination of CSF-to-serum albumin ratio (Q_Alb_) as indicative for BCSFB barrier function and correlation with regional BBB leakage (K^trans^).
- *Proteomic and transcriptomic analyses*: exploratory multi-omics approaches (e.g., targeted proteomics, RNA sequencing) to identify molecular signatures associated with BBB permeability.

Associations between biosample-derived markers and BBB permeability metrics (K^trans^) will be investigated using multivariable regression and mixed-effects models. Correction for multiple testing will be applied where appropriate. Integration of imaging and molecular data will be performed using hypothesis-driven and exploratory multivariate approaches. 

### Study progress and power analysis

An a priori power analysis was conducted to estimate the required sample size for the primary cross-sectional comparison of BBB permeability between individuals with SSD and HCs at baseline (V1). As prior DCE-MRI studies in schizophrenia report significant group differences in thalamic K^trans^ but do not provide standardized effect sizes, a moderate effect size was predefined for planning purposes (Cohen’s d = 0.47). This assumption was informed by DCE-MRI studies in ageing, mild cognitive impairment, and Alzheimer’s disease reporting effect sizes in the range of d ≈ 0.4–0.7 for BBB leakage metrics [42], as well as by reports of BCSFB dysfunction based on age-adjusted Q_Alb_ in SSDs [43]. Using a two-tailed independent-samples t-test with α = 0.05 and power (1–β) = 0.80, the required sample size was estimated at a minimum of *n* = 57 per group (total *N* = 114). Recruitment commenced in March 2023. As of March 2026, *N* = 118 individuals with SSD and *N* = 54 HC (total *N* = 172) have completed the baseline assessment (V1), thereby meeting the predefined requirements for the primary cross-sectional analysis and allowing additional power for within-SSD analyses. Initial results addressing the primary objective of baseline BBB permeability differences between SSD and HC have been published elsewhere and confirmed significantly increased BBB leakage in SSDs [44].

Following confirmation of baseline group differences, the study protocol was expanded to include the longitudinal follow-up assessments at 4–6 weeks after baseline (V2; early treatment phase) and at 2.5 years (V3; long-term outcome phase). Longitudinal analyses focus on within-subject changes in BBB permeability and are conducted using linear mixed-effects models.

Because no prior data are available on intra-individual correlations of BBB permeability metrics based on DCE-MRI across time in SSDs, longitudinal analyses are designed as a proof-of-concept at the current stage of the study. We therefore defined a pragmatic target of approximately *N* ≈ 30 SSD participants completing V1 and each follow-up visit (V2 and V3), balancing feasibility in a naturalistic clinical setting with the ability to detect moderate within-subject changes. Under a paired/repeated-measures framework (two-sided α = 0.05), *N* = 30 provides approximately 80% power to detect within-subject effects of d ≈ 0.53 or larger, while smaller effects may be underpowered and will be interpreted primarily via effect-size estimates and confidence intervals.

Expected attrition rates of approximately 15% at V2 and 30% at V3 were incorporated into the sampling strategy and closely match observed dropout rates. As V2 was implemented later (May 2025), an additional recruitment target of *N* = 35 SSD participants for V1 was defined to ensure approximately *N* ≈ 30 completers at V2, resulting in a revised target of *N* = 128 individuals completing V1. As of March 2026, *N* = 15 SSD participants have completed V2, and *N* = 20 have completed V3. The unequal number of completed V2 and V3 assessments reflects the chronological implementation of follow-up visits within the study protocol.

All power assumptions and sampling targets were cross-validated against prior longitudinal neuroimaging studies conducted at our site, reflecting a balance between statistical rigor, feasibility, and the constraints of a deeply phenotyped, naturalistic cohort.

#### Handling of missing data

Given the longitudinal and multimodal design, missing data are anticipated due to attrition, incomplete assessments, or technical factors (e.g., MRI quality control exclusions). Longitudinal analyses will primarily rely on linear mixed-effects models, which allow inclusion of participants with partially observed data and provide valid estimates under the assumption of data missing at random. The extent and patterns of missingness will be examined descriptively. Where appropriate, sensitivity analyses will be conducted to assess the robustness of findings to different assumptions regarding missing data. For DCE-MRI analyses, only datasets with complete acquisition and successful quality control will be included, as calculation of voxel-wise BBB permeability metrics (K_trans_) requires fully available imaging data. Participants with incomplete or technically insufficient DCE-MRI scans will therefore be excluded from Ktrans analyses, while remaining available data may be included in other analyses where appropriate.

### Outcomes and planned analyses

#### Primary outcome

The primary outcome is whole-brain voxel-wise blood–brain barrier (BBB) permeability quantified by DCE-MRI-derived transfer constant (K_trans_) at baseline (V1). The primary analysis compares mean K_trans_ values between individuals with SSD and HC.

#### Secondary outcomes

Secondary outcomes include:

- *Spatial leakage patterns*: regional K_trans_ differences (region-of-interest and voxel-wise analyses).
- *Longitudinal BBB dynamics*: within-subject changes in K_trans_ between V1, V2, and V3.
- *Clinical associations*: associations between BBB permeability metrics (K_trans_) and symptom severity (PANSS), global functioning (GAF, CGI), and cognitive performance (BACS, TMT, MoCA).
- *Biological correlates*: hypothesis-driven and exploratory associations between BBB permeability (regional K_trans_) and peripheral or CSF-based markers.
- *Exploratory stratification analyses*: identification of SSD subgroups based on BBB permeability profiles using data-driven clustering approaches.

All analyses are adjusted for relevant covariates including age, sex, body mass index, smoking status, and antipsychotic exposure where appropriate.

## Discussion

Psychiatry continues to face challenges in classifying mental diseases beyond symptom-based criteria, lagging behind fields such as oncology or rheumatology, where biomarker-guided treatment is already established [45]. The lack of validated biological markers remains a major barrier to implementing precision medicine in psychiatry [46]. Emerging evidence suggests that blood–brain barrier (BBB) dysfunction may represent a relevant biological mechanism in schizophrenia spectrum disorders (SSDs) and contribute to clinical heterogeneity, treatment response, and illness trajectory [47]. However, existing evidence is limited by indirect measures, small sample sizes, and predominantly cross-sectional designs.

The IMPACT study addresses these limitations by implementing a longitudinal DCE-MRI framework embedded in a deep phenotyping infrastructure. Prior work from our group demonstrates that integrating multimodal data can yield biological insights into SSD pathophysiology and establish associations with clinically relevant outcomes [48, 49]. The IMPACT protocol integrates quantitative BBB permeability mapping with standardized assessments of psychopathology, psychosocial functioning, and cognition, as well as repeated biosampling for multi-omics profiling. This facilitates the characterization of BBB leakage at clinically meaningful stages (acute psychosis, early treatment, long-term follow-up) and the investigation of cross-sectional and longitudinal associations with clinical and biological profiles. Crucially, the design facilitates analyses beyond case–control comparisons, allowing for the investigation of within-SSD heterogeneity and distinct subgroups. These methodological strengths position the study to support the evaluation of BBB-based stratification in SSDs.

Initial cross-sectional analyses [44] demonstrated increased BBB permeability in SSD compared with healthy controls, supporting feasibility and motivating the longitudinal extension. The longitudinal component is designed to determine whether BBB permeability exhibits state-related dynamics associated with symptom change and treatment, or whether it reflects a stable trait-like characteristic. Furthermore, the longitudinal structure enables future evaluation of whether baseline BBB permeability predicts subsequent clinical and functional outcomes, acknowledging that these analyses are exploratory at this stage.

Key methodological strengths of the study include: (i) repeated DCE-MRI within the same individuals across defined illness phases; (ii) harmonized multimodal MRI acquisition; and (iii) integration with comprehensive multimodal clinical phenotyping and biobanking.

Mechanistically, BBB alterations in SSD have been linked to inflammatory and vascular pathways [3, 6]. Meta-analytic evidence indicates elevated peripheral pro-inflammatory markers, including IL-6, TNF-α, and IL-1β, across SSD illness stages [21, 50, 51]. Furthermore, experimental work supports the link between inflammatory signaling and dysfunction in endothelial tight junctions and the neurovascular unit, which may increase barrier permeability [5].

Within this framework, IMPACT is positioned to evaluate hypothesis-driven associations, e.g., whether systemic inflammation relates to BBB leakage in a subgroup and whether BBB permeability metrics are associated with symptom dimensions, cognition, and longer-term functional trajectories.

In summary, IMPACT offers a structured, longitudinal methodology for quantifying BBB permeability in SSD using DCE-MRI and integrating BBB measures with in-depth clinical and biological phenotyping. The study aims to characterize the temporal dynamics of blood–brain barrier (BBB) dysfunction in schizophrenia spectrum disorders (SSD) and to evaluate its clinical and biological relevance. In addition, it seeks to establish a robust data foundation for biomarker-guided stratification and the development of mechanism-driven hypotheses within precision psychiatry.

**Table 1:** Overview of baseline and follow-up examinations

	Study visit		
	Day 0 (baseline)	Day 28 − 14 days	V1 + 2.5 years (+/- 90 days)
Assessments	V1	V2	V3
Clinical characterization			
Basic phenotyping Munich Mental Health Biobank and German Center for Mental Health (DZPG)	X		
M.I.N.I. interview (DSM-5-TR)	X		
Check inclusion/exclusion criteria	X	X	X
Psychiatric and medical history	X	X	X
Previous and current use of alcohol and illicit drugs	X	X	X
Current and previous medication	X	X	X
PANNS	X	X	X
PANNS RSWG criteria	X	X	X
CDSS	X		
CGI-S	X	X	X
GAF	X	X	X
Biomaterials			
Biobank blood sampling	X		
Cerebrospinal fluid (optional)	X		
Serum	X	X	X
Neuroimaging			
sMRI, rsfMRI, ASL	X	X	X
DCE-MRI	X	X	X

The basic phenotyping of the Munich Mental Health Biobank has been described in detail elsewhere [26].* ASL* arterial spin labeling;* BACS* brief assessment of cognitive function in Schizophrenia;* CDSS* calgary depression scale for Schizophrenia;* CGI* clinical global impression;* DCE* dynamic contrast-enhanced;* GAF* global assessment of functioning;* PANNS* positive and negative syndrome scale;* rsfMRI* resting state functional MRI;* sMRI* structural MRI;* TMT* trail making test

## Data Availability

No datasets were generated or analysed during the current study.
